# Reflections about attendance in the Norwegian cervical cancer screening program among the youngest invited women aged 25–29 years: a qualitative interview study

**DOI:** 10.1177/14034948251393105

**Published:** 2025-11-24

**Authors:** Marit Solbjør, Marthe Hagen, Iren Harstad, Ingrid Baasland

**Affiliations:** 1 Department of Public Health and Nursing, Norwegian University of Science and Technology, Trondheim, Norway; 2 Department of Public Health and Nursing, General Practice Research Unit, Norwegian University of Science and Technology, Trondheim, Norway; 3 Norwegian Cervical Cancer Screening Program, Cancer Registry of Norway, Norwegian Institute of Public Health, Oslo, Norway

**Keywords:** HPV, screening, cervical cancer, participation, women, Norway, qualitative

## Abstract

**Aims:**

Understanding motivators for cervical cancer screening among young women is critical for improving participation rates and reducing the incidence of cervical cancer. Young women have low participation rates in the CervicalScreen Norway. In 2022, the first cohort of school-based human papilloma virus vaccinated women entered the screening programme at the age of 25 years. This paper investigates reflections regarding cervical cancer screening and factors that influence attendance among first or second-time invitees in the CervicalScreen Norway.

**Methods:**

The study comprises 18 qualitative semi-structured individual interviews with Norwegian women aged 25–29 years of which 14 had attended screening. We used reflexive thematic analysis with a critical approach and identified three main themes related to trust, social expectations, and perceived vulnerability.

**Results:**

The three main themes were ‘trusting health authorities but wishing for a clearer message’; ‘normalisation of screening participation driven by social expectations’; and ‘efforts to avoid vulnerability during the test procedure’. Trusting the authorities to provide beneficial health initiatives, the women would follow advice about screening. Social expectations led to consider screening participation as the right thing to do. These young women felt vulnerable and inexperienced with gynaecological examinations and opted for screeners that reduced their feeling of vulnerability.

**Conclusions:**

**Providing clear information on the screening procedure and expectations during gynaecological examinations is essential to reduce vulnerability and enhance participation among young women. Communication should highlight that screening is readily accessible in primary healthcare and be shared through media platforms popular with younger women.**

## Introduction

Cervical cancer is the fourth most common cancer among younger women and is caused by human papilloma virus (HPV) in 99% of cases [1]. HPV is transmitted through sexual contact and has a high prevalence in the population. Around 90% of these infections regress or are not detectable after 2–3 years and will never develop into cancer, but some persistent infections might lead to premalignant lesions and further on to cancer if not treated [2,3]. Two strategies exist to prevent cervical cancer: vaccination and screening. School-based HPV vaccination has been implemented in many countries, including Norway, during the past decade. It has been demonstrated to be highly effective in preventing infection with high-risk HPV [4]. Although HPV vaccination reduces cervical cancer risk, screening remains essential to detect and treat precancerous lesions. Understanding how HPV-vaccinated women perceive screening is critical to improving participation rates. Through screening, either HPV or cytology based, abnormal cervical tissue can be detected and removed before developing into cancer. Screening can reduce the development of cervical cancer by over 70% [5,6]. The age to start screening differs in the Nordic countries: at 30 years in Finland, at 23 years in Sweden, Denmark, and Iceland, and at 25 years in Norway. Awareness of HPV has increased following the implementation of HPV vaccination, but over 24% of Scandinavian women had not heard about HPV in 2017 [7]. Among the screening population in Norway aged 25–69 years, 66% participate, but participation rates vary across age groups – with the youngest having the lowest participation rate [8]. Previous research has identified several reasons for participation, including the opportunity to prevent cancer through the early detection of abnormal cells [9,10] or seeing oneself as at risk [11]. Perceiving a low personal risk of the disease may result in non-participation [12,13]. HPV-vaccinated women in Denmark saw themselves as having a higher risk for HPV than unvaccinated women did, and were more inclined to intend to participate in screening than unvaccinated women [14]. In the UK, unvaccinated women were also less likely to attend cervical cancer screening [15], indicating a social gradient in utilisation of public health services. However, a Danish study found that some HPV-vaccinated women were uncertain about the aim of screening when being vaccinated [16]. This uncertainty may lead them to view screening as unnecessary. Knowledge is, however, limited about how young, HPV-vaccinated women in Norway reflect about participation in HPV screening. The HPV vaccination coverage among 7th grade girls in the school-based programme has risen from 67% during the initial years to 93% in 2023 [17]. In 2022, the youngest cohort invited to CervicalScreen Norway (CSN) at the age of 25 years, was the 1997 birth cohort, all of whom had been offered school-based HPV vaccination at age 12–13 years. Women born in 1991 and later were offered HPV vaccination as part of a catch-up public health initiative in 2016–2019. It is vital to gain a deeper understanding of how HPV-vaccinated women in Norway perceive the benefits of screening and what influences their decision to participate. This article explores how these youngest women, aged 25–29 years, reflect on cervical cancer screening.

## Methodology

This was a qualitative study with individual interviews that investigate young women’s views on participating in cervical cancer screening. Individual qualitative interviews are particularly suitable for examining sensitive subjects. Cervical cancer screening can be seen as sensitive because the screening test detects sexually transmitted HPV and the screening test is done at a gynaecological examination. This study uses data from two master theses in public health from NTNU, which have been combined and re-analysed through a secondary analysis with analytic expansion [18].

### Setting

Women aged 25–69 years are invited to attend the CSN at regular intervals. They must book an appointment for the screening test themselves, mainly at the general practitioner (GP) or gynaecologists or midwifes. HPV testing became the primary screening method for all age groups in the CSN in July 2023. Prior to 2023, the youngest women were offered cytology-based screening. Therefore, the women participating in the current study in 2022 and early 2023 were not screened for HPV but were offered cervical smears. Women born in 1997 or later have been offered HPV vaccination in the school-based vaccination programme at the age of 12 years. Women born in 1992–1997 were offered catch-up vaccination at an older age through a special initiative.

### Recruitment

Recruitment to the study was done through information leaflets at a university campus and a waiting room at a gynaecological practice; social media; and snowball sampling. Snowball sampling was done by authors MH and IH, who at the time were students aged 22 and 25 years. They used their extended social network to spread information about the project. Individuals who were directly acquainted with the researchers were not included in the study.

### Sample

Study participants comprised 18 women born in 1992–1997. Sixteen were HPV vaccinated, two did not reveal their vaccine status. Three of the women had planned to participate in the CSN, 14 had participated, while one participant would not participate until later in life. Eleven participants were students, of which eight were doing master’s degree level. Five women were studying or had completed health sciences education. Seven women were employed, of which two worked within the health services. Two participants had children. All the women spoke Norwegian fluently and lived in Norway.

### Data collection and analysis

Eighteen individual semi-structured interviews were carried out by authors MH and IH, either face to face, or digitally on teams or zoom. Each interviewer used an interview guide to structure the conversation. The interviews lasted from 16 to 35 minutes. All interviews were audio recorded and transcribed verbatim.

Analysis was done with reflexive thematic analysis (RTA), which is a six-step process including coding, interpretation and development of themes [19]. The primary analysis was done separately on each of the two datasets using semantic coding with an experiential approach. Across the two datasets, the primary analysis gave ten experiential themes, including ‘cervical test is prioritised’, ‘relations to family and friends contributes to participation’, ‘information on the internet contributes to knowledge about cervical cancer screening’, ‘health personnel are important’, ‘trusting recommendations from the authorities’, ‘the screener’s competence and gender is important’, and ‘information sources are important for participation’. The secondary analysis used RTA with a critical approach [19]. We compared themes from the two primary analyses to find patterns across the full dataset. Then we re-read all interviews and coded them with latent codes, using trust and vulnerability as sensitising concepts. Following steps four and five of RTA [19], we identified initial themes related to trust in medical doctors, undergoing gynaecological examinations, and sources of information. These were evaluated and discussed by all authors before constructing the three final themes.

### Ethics

The two studies were approved by Sikt (reference no. 503290 and 379161). All participants received written and oral information and signed an informed consent form. Audio recordings were stored in NICE-1 (demands two-factor authentication) and were later deleted at the preset date.

## Results

We identified three primary themes: trusting health authorities but wishing for a clearer message; normalisation of screening participation driven by social expectations; and efforts to avoid vulnerability during the test procedure.

### Trusting health authorities but wishing for a clearer message

These women had received two public health offers to prevent cervical cancer: first the HPV vaccine, and later the screening programme. Trusting public health initiatives from the health authorities, their parents or themselves had chosen that they should be vaccinated. They trusted that cervical cancer screening was a beneficial initiative from the health authorities. Their trust was built on a generalised trust in vaccination and medical testing, and that the health authorities worked for the benefit of the population, as described by Ingrid:

I have a very high trust in that. . . when the state and everybody says that you need to do this to make things better, I’ll just do it. (Ingrid)

The connection between these two preventive initiatives were understood in different ways. Being uncertain about the effect of the vaccine led vaccination to be of less importance in their decision making on participating in screening. Some believed that they had been part of a vaccine trial, others knew HPV vaccination was part of the public vaccination programme but did not trust it to prevent all cervical cancers. Therefore, they wanted to have a cervical test. Not all knew the word screening and referred to the vaccination programme when asked about the ‘cervical cancer screening program’. The participants knew, however, that women in Norway are invited to attend cervical cancer testing from age 25 years.

All the study participants would have been sent information about the screening programme when turning 25 years, but not all remembered receiving it. Having signed up for digital post from public services could lead to overlooking the invitation because it arrived as one email among many. For those who remembered the letter from the screening programme, it was a reminder about the importance of cervical cancer screening. Ida understood the information as a message that she ought to participate in screening:

Eh, I think I got one [an invitation]. I got one saying you ought to go. But I don’t know if. . . There was no appointment, as I remember it. I can’t really remember. But I had received some information in advance, so I had got an initiative to get it done. (Ida)

But despite seeing the invitation letter as a nudge towards participation, not receiving a pre-set appointment led to questions about the priorities of the health authorities. For them, a preset appointment would have emphasised the importance of the test. Therefore, leaving the planning of the test to each woman was interpreted as health authorities not seeing cervical cancer screening as important enough to prioritise it. This led these young women to call for an even clearer message about the importance of cervical cancer screening.

### Normalisation of screening participation driven by social expectations

For these young women, the existence of a national screening programme suggested that participation was as the right thing to do. For some, it was enough to know that screening was recommended by the health authorities. Others looked for more information than what was provided through the invitation. They found the link to the programme webpage helpful to find more information, but also found information through mass media, social media, and their social network, which all contributed to pro-screening discourses. For Ida, participation was a normal part of the health repertoire when entering the relevant age group:

I knew well about it in beforehand. Because there’s been a lot of media coverage on the subject, and lots of talk about it. At least in channels where I participate. So having a Pap smear at 25 was kind of natural. (Ida)

Friends and family expected women to participate. Mothers and sisters encouraged the younger women to participate. Friends talked about experiences with cervical testing and the benefit of participating in screening. These conversations provided social pressure to participate, constructing non-participation as deviant, as explained by Julie:

The first time I had a Pap smear was because my friends had done it. That made me, well, it made me feel that I ought to do it too, right? Because you don’t want to be the one person who hasn`t done it. (Julie)

Stories from family and friends who had experienced having abnormal cells of the cervix or treatment (conisation) because of such abnormalities, led to seeing the screening programme as beneficial. Early detection was also important due to other cancer narratives within their families. These women experienced that frightening cancer stories led to worries which could be solved through participation in the cervical cancer screening programme. But social expectances of participation could be experienced as an impediment to making an individual informed choice. The strength of the pro-screening discourse was particularly evident for the one woman who had decided to delay participation. She experienced the information in the invitation letter as a strategy to scare women to participate in the programme:

So, it is a bit like – okay, they choose statistics that look most frightening to make us participate in this programme. (Anna)

In social media, it was stories about young women who had cervical cancer that accentuated the importance of testing. Such stories were also present as documentaries or news stories and were seen as important for awareness of women’s health. However, these stories were frightening and interpreted as strategies to scare women to participate in screening. Seeing success stories about cervical cancer screening in which women were saved due to early detection of abnormal cells, or about women having a negative test, was wanted to understand that cervical cancer could be prevented.

### Efforts to avoid vulnerability during the test procedure

These women were worried about being vulnerable when participating in cervical cancer screening. Vulnerability included undergoing a gynaecological examination and fear of having cervical cancer. Little or no experience with gynaecological examinations led to uncertainty about what would happen during the screening test. Not all the women knew how the test was performed and if it was painful. One woman believed that the screening test included a biopsy and was relieved after her first screening test. Those who had experienced a screening test thought it was quick and simple. Even when unpleasant, the test would not deter them from continue participation if they could feel in control during the test procedure.

Avoiding vulnerability would be solved through choosing who should conduct the test, although they varied in their opinions of who they preferred. Not all women knew that their GP was the primary tester for the cervical cancer screening programme, which sometimes led to confusion about booking an appointment for a screening test. Three elements were important for the women when choosing their screening provider: competence, gender, and familiarity. Competence in doing the test was an important factor for whom they would prefer to do the test. Seeing the gynaecologist as having more specialised competence led some women to prefer having the test there, while others trusted the GP’s competence. The gender of the screening provider could also influence vulnerability. For some, having a male doctor to do the gynaecological examination was difficult because he represented men in heterosexual relationships. A female doctor felt less vulnerable because she was expected to understand better how a woman would feel during the gynaecological examination.

It would be more uncomfortable and would be more, well, unfamiliar and weird. Because I’m attracted to men, and in other settings, eh. . . undressing is not for having a [medical] test, you know. (Silje)

A female doctor felt less vulnerable because she was expected to understand better how a woman would feel during the gynaecological examination. Also, female screeners were expected to be more familiar with the female anatomy.

But when the female parts are involved, you think that doctors who have the same parts maybe know best or take it as serious as yourself. (Maria)

But showing their vagina to another person could be difficult regardless of gender. One strategy was to do the screening test with someone whom they were certain would not see their body as unusual. Some felt less vulnerable being one of many women who undressed in front of a gynaecologist than in front of a GP who did these examinations more rarely. Another aspect of this was to separate their gynaecological issues from other parts of their lives. A gynaecologist would only know about their gynaecological issues, while having the GP examining their vagina as well as knowing so many aspects of their health and life could feel intimidating.

It feels safe in a way because you can build a relationship with your GP. That is, I feel I can trust gynaecologists as much, but with them you just visit a random one, unless you know that gynaecologist beforehand. (Malin)

However, the women varied in how they connected familiarity and vulnerability. Some felt safe to do the cervical screening test with their GP who knew them well because they were seen as a whole person. Regardless of who they preferred as their screener, the gynaecological examination was associated with exposure and vulnerability. What varied was how they solved this vulnerability when choosing where to have the cervical cancer screening test.

## Discussion

### Trust in health authorities and the normalisation of screening

In the current study, women who had been offered HPV vaccination and later were invited to attend cervical cancer screening reflected on screening through their trust in health authorities, normalising discourses, and attempting to avoid vulnerability during gynaecological examinations. Previous research suggests that HPV-vaccinated women can be uncertain about the purpose of screening [16]. In our study, the relationship between HPV vaccination and screening was unclear. They did not perceive being HPV vaccinated as a guarantor against cervical cancer, and therefore saw screening as an important health initiative. Consistent with research from Denmark and the UK [14,15] showing that women who are HPV vaccinated have higher participation rates in screening than non-vaccinated women, participants in our study were positive to both initiatives. Our participants had high education, suggesting similar associations between participation and high socioeconomic status as in other countries [14,15]. However, HPV vaccination rates in Norway are currently at 93% [17] indicating that participation in vaccination and screening have different explanations. In our study, trusting health authorities to provide valuable health initiatives led to participation without further evaluation. Hofmann and Stanik argue that preset appointments is a nudge that hinders informed choice in screening [20]. But in our study, not receiving a preset appointment appeared paradoxical when recommending screening through a public programme. The invitation to begin screening did, however, create a notion of what invitees ought to do, thereby normalising participation. Consistent with other studies [9,16,21], women in our study were more likely to participate if they know someone who has participated, and participation was also normalised through social media campaigns and social expectations.

### Overcoming vulnerability facilitates screening participation

As women in the present study were first or second-time invitees to cervical cancer screening, they raised questions about how the examination was done. Not all women aged 25 years are familiar with gynaecological examinations. Consistent with previous studies [11,22], our findings highlight that vulnerability during gynaecological examinations remains a potential barrier to participation. Having a male doctor [8] or hearing stories about unpleasant screening experiences [9] can lead to avoiding the cervical screening examination. Women in the present study had strategies to diminish vulnerability through choosing their screener. More knowledge about the examination itself could help women who are new to screening to trust the competence of different screeners.

### Future directions: HPV self-sampling

A future solution is HPV self-sampling, in which women obtain the screening test themselves with a cotton swab, and could solve vulnerability during testing. Self-sampling is currently being initiated or tested in several countries, including Norway. However, it creates new demands for sufficient information from screening providers to ensure an informed choice in the participants when testing is done without seeing a health professional.

### Strengths and weaknesses

This is the first study to investigate important issues for the youngest invitees in the cervical cancer screening programme in Norway. The secondary analysis was based on two studies with slightly different aims and interview guides, which led to some issues being more developed in one of the data materials than in the other. However, similar stories were told across the two data sets. This could be caused by the recruitment method, with snowballing leading to a selection bias of women who have adjacent social networks, therefore telling similar stories that relate to a dominant discourse on cervical cancer screening. Also, participants all had higher education and several had professional health competence, which might have influenced their knowledge about health issues compared with other women their age.

## Conclusions

Providing information about how the screening test is conducted and what to expect during a gynaecological examination is crucial for reducing feelings of vulnerability and encouraging screening attendance among younger women. This information should emphasise that the screening test is easily carried out in primary healthcare settings. Additionally, communication about cervical cancer screening should be delivered through media platforms commonly used by younger women. Enhancing participation in cervical cancer screening among young women is critical for the long-term success of public health initiatives aimed at reducing cervical cancer.

## References

[1] Walboomers JM Jacobs MV Manos MM , et al. Human papillomavirus is a necessary cause of invasive cervical cancer worldwide. J Pathol 1999;189:12–19. 10.1002/(SICI)1096-9896(199909)189:1<12::AID-PATH431>3.0.CO;2-F 10451482

[2] Winer RL Hughes JP Feng Q , et al. Early natural history of incident, type-specific human papillomavirus infections in newly sexually active young women. *Cancer Epidemiol, Biomarkers Prevent*: a publication of the American Association for Cancer Research, cosponsored by the American Society of Preventive Oncology 2011;20:699–707. 10.1158/1055-9965.EPI-10-1108 PMC3078690 21173170

[3] Rodriguez AC Schiffman M Herrero R , et al. Longitudinal study of human papillomavirus persistence and cervical intraepithelial neoplasia grade 2/3: critical role of duration of infection. J Natl Cancer Inst 2010;102:315–324. 10.1093/jnci/djq001 PMC2831050 20157096

[4] Kavanagh K Pollock KG Cuschieri K , et al. Changes in the prevalence of human papillomavirus following a national bivalent human papillomavirus vaccination programme in Scotland: a 7-year cross-sectional study. Lancet Infect Dis 2017;17:1293–1302. 10.1016/S1473-3099(17)30468-1 28965955

[5] Vaccarella S Franceschi S Engholm G , et al. 50 Years of screening in the Nordic countries: quantifying the effects on cervical cancer incidence. Br J Cancer 2014;111:965–969. 10.1038/bjc.2014.362 PMC4150271 24992581

[6] Lonnberg S Hansen BT Haldorsen T , et al. Cervical cancer prevented by screening: long-term incidence trends by morphology in Norway. Int J Cancer 2015;137:1758–1764. 10.1002/ijc.29541 25833121

[7] Thomsen LT Nygard M Stensen S , et al. Awareness of human papillomavirus after introduction of HPV vaccination: a large population-based survey of Scandinavian women. *Eur J Cancer Prevent*: the official journal of the European Cancer Prevention Organisation (ECP) 2017;26:170–178. 10.1097/CEJ.0000000000000238 27010329

[8] Leinonen MK Campbell S Klungsoyr O , et al. Personal and provider level factors influence participation to cervical cancer screening: a retrospective register-based study of 1.3 million women in Norway. Prev Med 2017;94:31–39. 10.1016/j.ypmed.2016.11.018 27894911

[9] Chorley AJ Marlow LA Forster AS , et al. Experiences of cervical screening and barriers to participation in the context of an organised programme: a systematic review and thematic synthesis. Psychooncology 2017;26:161–172. 10.1002/pon.4126 PMC5324630 27072589

[10] Wilding S Wighton S Halligan D , et al. What factors are most influential in increasing cervical cancer screening attendance? An online study of UK-based women. Health Psychol Behav Med.2020;8:314–328. 10.1080/21642850.2020.1798239 PMC8114340 34040874

[11] Aasbo G Solbraekke KN Waller J , et al. Perspectives of non-attenders for cervical cancer screening in Norway: a qualitative focus group study. BMJ Open 2019;9:e029505. 10.1136/bmjopen-2019-029505 PMC6719771 31471438

[12] Hendry M Pasterfield D Lewis R , et al. Are women ready for the new cervical screening protocol in England? A systematic review and qualitative synthesis of views about human papillomavirus testing. Br J Cancer 2012;107:243–254. 10.1038/bjc.2012.256 PMC3394982 22699825

[13] Iyer AL Bundorf MK Gyrd-Hansen D , et al. How does information on the harms and benefits of cervical cancer screening alter the intention to be screened?: a randomized survey of Norwegian women. *Eur J Cancer Prevent*: the official journal of the European Cancer Prevention Organisation (ECP) 2019;28:87–95. 10.1097/CEJ.0000000000000436 PMC6160367 29595751

[14] Hestbech MS Gyrd-Hansen D Kragstrup J , et al. How does HPV vaccination status relate to risk perceptions and intention to participate in cervical screening? a survey study. BMC Public Health 2016;15:708. 10.1186/s12889-016-3397-y PMC4973036 27488178

[15] Beer H Hibbitts S Brophy S , et al. Does the HPV vaccination programme have implications for cervical screening programmes in the UK? Vaccine 2014;32:1828–1833. 10.1016/j.vaccine.2014.01.087 PMC3991313 24530938

[16] Mortensen JH Bigaard J Kvernrod AB. Young Danish HPV vaccinated women’s knowledge, barriers and facilitators towards cervical cancer screening: a qualitative study. Prev Med Rep 2021;24:101507. 10.1016/j.pmedr.2021.101507 PMC8365507 34430190

[17] NIPH. Vaccination for Human Papillomavirus (HPV) (Indicator 22) Oslo: Norwegian Institute of Public Health; 2025 [updated 23.01.2025]. www.fhi.no/en/nc/Indicators-for-NCD/vaccine-and-screening/hpv-vaccine-indicator-22/ (accessed 27 October 2025).

[18] Thorne S. Secondary qualitative data analysis. In: Beck CT (ed.) Routledge International Handbook of Qualitative Nursing Research. New York: Routledge; 2013. pp. 393–404.

[19] Braun V Clarke V. Thematic Analysis. A Practical Guide. London: Sage; 2022.

[20] Hofmann B Stanak M . Nudging in screening: Literature review and ethical guidance. Patient Educ Counseling 2018 101(9):1561-1569. doi: 10.1016/j.pec.2018.03.021. Epub 2018 Mar 27. PMID: 29657111. 10.1016/j.pec.2018.03.021 29657111

[21] Arabaci Z Ozsoy S. The pap-smear test experience of women in Turkey: a qualitative study. Asian Pacific J Cancer Prevent: APJCP 2012;13:5687–5690. 10.7314/apjcp.2012.13.11.5687 23317239

[22] Larsen M Oldeide CC Malterud K. Not so bad after all . . ., women’s experiences of pelvic examinations. Fam Pract 1997;14:148–152. 10.1093/fampra/14.2.148 9137954

